# 

**Published:** 1894-11-10

**Authors:** 


### CONTENTS.

**The Pay System and the Profession** - 91
**Inheritance** - 92
**The Study of Man. VIII.**—Language and Race - 93
**Medical Progress and Hospital Clinics:**
The Value of Optic Neuritis in Medical Diagnosis - 97
Muscular Rheumatism - 98
Progress in Medicine.—Diseases of the Heart and Vascular System (*continued*) - 99
Progress in Surgery.—Surgery of the Intestines - 100
Periscope of Dermatology - 101
New Drugs and Preparations - 102
**Studies in Applied Sociology.**—Are Suicides Mad? - 94
**Annotations.** — Tuberculosis in Cattle—Infantile Scurvy — The Public and the London Medical Schools - 95
**Notes and News** - 96
**The Institutional Workshop:**
Within the Hospitals.—Devon and Exeter Hospital - 103
Foreign Hospitals - 104
Practical Departments - 104
Constuction Notes - 105
**Editor's Letter Box** - 106
**The Book World of Medicine and Science** - 107

### EXTRA SUPPLEMENT.—THE NURSING MIRROR.

**News from the Nursing World.**—Our Princess—The Queen of Greece—Wandering Nurses—Ambulance Work—Male Nurses in the Army—Representative or Registered?—Where was the Doctor?—Addenbrooke's Hospital—"Born to Measles"—Staleybridge—Paris—Danger Signals—Teachers and Taught—Private Nurses in South Wales—The Profits of Private Nurses—Friedenheim—Short Items - xxxv

**Notes from St. Helena—Nurses at Rio de Janiero** - xxxvii

**Death in Our Ranks—Appointments** - xxxvii

**News from Victoria** - xxxviii

**The Glasgow System for Probationers** - xxxix

**Presentations** - xxxix

**The Matrons' Council** - xxxix

**Where to Go** - xxxix

**Notes and Queries** - xxxix

**Dress and Uniforms—Everybody's Opinion** - xl

